# CRISPR Turbo Accelerated KnockOut (CRISPy TAKO) for Rapid *in vivo* Screening of Gene Function

**DOI:** 10.3389/fgeed.2020.598522

**Published:** 2020-10-21

**Authors:** Sonja L. Plasil, Amit Seth, Gregg E. Homanics

**Affiliations:** 1 Department of Pharmacology and Chemical Biology, University of Pittsburgh School of Medicine, Pittsburgh, PA, United States; 2 Department of Anesthesiology and Perioperative Medicine, University of Pittsburgh School of Medicine, Pittsburgh, PA, United States; 3 Department of Neurobiology, University of Pittsburgh School of Medicine, Pittsburgh, PA, United States

**Keywords:** CRISPR/Cas9, genome editing, ethanol, increased mutagenesis, accelerated phenotypic screening, functional knockout

## Abstract

The development of CRISPR/Cas9 technology has vastly sped up the process of mammalian genome editing by introducing a bacterial system that can be exploited for reverse genetics-based research. However, generating homozygous functional knockout (KO) animals using traditional CRISPR/Cas9-mediated techniques requires three generations of animals. A founder animal with a desired mutation is crossed to produce heterozygous F1 offspring which are subsequently interbred to generate homozygous F2 KO animals. This study describes an adaptation of the CRISPR/Cas9-mediated method to develop a cohort of homozygous gene-targeted KO animals in one generation. A well-characterized ethanol-responsive gene, *MyD88*, was chosen as a candidate gene for generation of KO mice as proof-of-concept. Previous studies have reported changes in ethanol-related behavioral outcomes in MyD88 KO mice. One-cell mouse embryos were simultaneously electroporated with four gRNAs targeting a critical Exon of MyD88 along with Cas9 protein. DNA and RNA analysis of founder mice revealed a complex mix of genetic alterations, all of which were predicted to ablate MyD88 gene function. Behavioral testing confirmed the hypothesis that successful one-generation KO of MyD88 would reproduce the decreased ethanol-induced sedative/hypnotic effects and increased ethanol consumption in males that were observed in previous studies. This study additionally compared responses of Mock treatment control mice generated through electroporation to controls purchased from a vendor. No substantial behavioral changes were noted between control cohorts. Overall, the CRISPR/Cas9 KO protocol reported here, which we call CRISPR Turbo Accelerated KnockOut (CRISPy TAKO), will be useful for reverse genetic *in vivo* screens of gene function in whole animals.

## INTRODUCTION

Clustered regulatory interspaced short palindromic repeats (CRISPR) paired with CRISPR associated protein 9 (Cas9) is currently the dominant and preferred gene editing tool in scientific research. CRISPR-based screens of gene function *in vitro* have been tremendously useful for identifying genes involved in tumor suppression (Michels et al., 2020), mitochondrial function (Khan et al., 2020), and dendritic development (Muir et al., 2020). High throughput CRISPR loss-of-function reverse genetic screens allow for the rapid identification of genes involved in phenotypes of interest. However, *in vitro* screens are limited by phenotypes that can be readily assayed in cell culture, (e.g., cellular proliferation, drug sensitivity, and cell survival). Further, the acquisition of transcriptome data has greatly outpaced our capacity to functionally study genes of interest. For many biological questions, particularly those that pertain to dysfunction of the central nervous system where behavioral abnormalities are the primary phenotype of interest, *in vivo* tests of behavior must be employed. Because behavior is the phenotype of interest, *in vitro* screens are unsatisfactory. In this study, we sought to develop a method with moderately high throughput that could be used *in vivo* to screen genes for effects on behavior.

**FIGURE 1 |:**
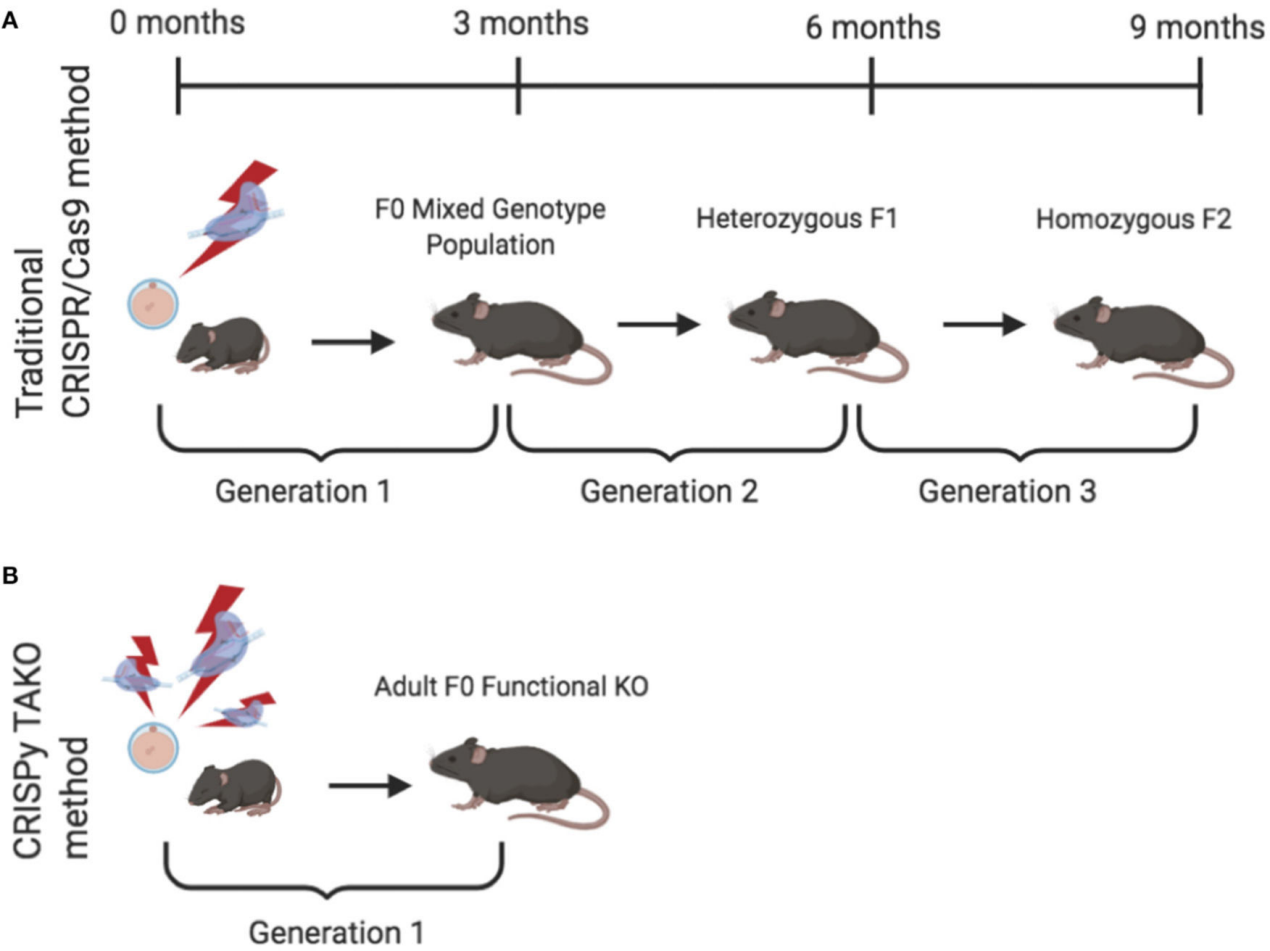
Comparison of the timeline required to produce knockout (KO) mice for behavioral testing using the traditional and CRISPy TAKO approaches. **(A)** Traditional CRISPR/Cas9-mediated method to create a stable KO line. Founder (F0) animals are an eclectic mix of wild-type (WT), heterozygous, and homozygous KOs. A founder with an inactivating mutation is selected for breeding to establish a KO line of mice. First generation offspring (F1) are heterozygous and must be interbred to produce an F2 generation. A subset (~25%) of the F2 generation are homozygous KO mice and can be compared for behavioral phenotypes with WT littermates. **(B)** CRISPy TAKO method for creating functional KO mice. By using multiple gRNAs that target a small but functionally critical part of the gene of interest, most F0 mice harbor biallelic mutations that functionally inactivate the gene of interest and are suitable for behavioral phenotyping.

Global gene knockout (KO) animal models are a gold standard approach that have been widely used to study and delineate the effects of individual molecules in whole organisms. The recent application and widespread adoption of CRISPR/Cas9 technology dramatically facilitated KO animal generation. However, the standard method of creating CRISPR KO animals, a.k.a., CRISPy Critters (Homanics, 2019), typically requires three generations to produce experimental animals that can be phenotypically evaluated and therefore is unsuitable for moderate-high throughput *in vivo* screens (Figure 1A). Briefly, in a typical CRISPR KO animal study, CRISPR reagents are introduced to one-cell embryos that develop into founder (F0) animals that are screened for the desired mutation. F0 animals are typically an eclectic mix of wild-type (WT) and mutant animals. The mutants may be heterozygotes, homozygotes, or compound heterozygotes, and most mutant alleles differ in the individual mutations they harbor in the target gene of interest. A founder animal that harbors a desirable mutation (typically a frameshift or a large deletion) is then mated to WT mice to produce heterozygous F1 offspring. Subsequently, heterozygotes are interbred to produce homozygous F2 mutant KO offspring. These F2 mutant animals have both alleles of the gene of interest inactivated, they all harbor the same mutation in the gene of interest, and they can be compared to WT littermate controls for relevant phenotypic changes. Although this CRISPR approach for creating gene KO animals represents a dramatic savings in time, effort, and expense compared to traditional embryonic stem cell-based gene targeting approaches, the CRISPR approach still requires considerable time and expense because three generations of animal production is time consuming and results in substantial animal care and housing expenses. This process also requires a considerable amount of personnel time for colony maintenance and genotyping.

We endeavored to establish a one-generation CRISPR KO approach in which F0 animals could be directly used to test for the behavioral consequences of gene inactivation. We reasoned that a very high efficiency CRISPR mutagenesis approach could be used to efficiently create F0 animals in which both alleles of the gene of interest are mutated and are functionally inactivated (i.e., gene KOs) (Figure 1B). Although each F0 animal may have different mutations, they would all be functionally and phenotypically equivalent if a critical part of the gene were sufficiently mutated.

Our long-term goal is to employ this accelerated technique to vastly speed up the screening process of testing novel ethanol-responsive genes for involvement in ethanol-related behavioral phenotypes, including ethanol consumption. Therefore, we initially piloted this approach *in vitro* on two novel ethanol-responsive long non-coding RNA (lncRNA) genes (*4930425L21Rik* and *Gm41261)*. We subsequently sought to validate this method *in vivo* by mutating a gene previously shown to alter behavioral responses to ethanol when inactivated using traditional global KO technology. *MyD88* was chosen as a well-characterized ethanol-responsive gene for proof-of-concept as prior studies have evaluated the effects of MyD88 global KO on ethanol-related behaviors, including ethanol drinking (Blednov et al., 2017b) and response to ethanol’s acute sedative/hypnotic and motor ataxic effects (Wu et al., 2012; Blednov et al., 2017a). Single generation F0 MyD88 KO animals were hypothesized to exhibit decreased ethanol-induced sedative/hypnotic effects, decreased sensitivity to ethanol-induced motor ataxia, and a male-specific increase in ethanol consumption relative to controls.

To further streamline this accelerated KO mouse protocol, we reasoned that for first pass screening of genes for behavioral phenotypes, isogenic animals purchased directly from a vendor could be used as a control group for comparison to KOs. However, one concern is that the CRISPR and/or electroporation procedures, irrespective of the gene being mutated, could hypothetically exert uncharacterized deleterious effects. Therefore, we also created in-house Mock treatment controls that were produced under an identical protocol to the KOs except that the Mock-treated animals were created with procedures that lacked custom CRISPR RNA (crRNA). This Mock-treated control group was directly compared to isogenic C57BL/6J WT mice (Jax controls) purchased from the Jackson Laboratory (JAX). We hypothesized that these two control groups would not differ on behavioral endpoints of interest.

In this report, we describe implementation and validation of a novel technique for the accelerated production of CRISPR KO mice in one generation. Animals produced via this protocol are herein affectionately referred to as CRISPR Turbo Accelerated KnockOuts (i.e., CRISPy TAKOs). We report that our CRISPR protocol can reliably produce a large number of F0 KO animals and that the ethanol phenotype of MyD88 CRISPy TAKOs largely recapitulates results previously reported for traditional MyD88 global KOs. Furthermore, for the behaviors tested in this study, vendor purchased mice and Mock treatment controls did not differ substantially. Together, these results establish the CRISPy TAKO method for screening gene function *in vivo*. This method has moderately high throughput and will be especially useful for phenotypes, such as behavioral responses, that cannot be assayed *in vitro*.

## MATERIALS AND METHODS

### Animals

All experiments were approved by the Institutional Animal Care and Use Committee of the University of Pittsburgh and conducted in accordance with the National Institutes of Health Guidelines for the Care and Use of Laboratory Animals. C57BL/6J male and female mice used to generate embryos for electroporation and the purchased control group were procured from The Jackson Laboratory (Bar Harbor, ME). CD-1 recipient females and vasectomized males were procured from Charles River Laboratories, Inc. (Wilmington, MA). Mice were housed under 12-h light/dark cycles, with lights on at 7 a.m. and had *ad libitum* access to food (irradiated 5P76 ProLab IsoProRMH3000; LabDiet, St. Louis, MO) and water.

### gRNA Design

Guide RNAs (gRNAs) were generated using a commercially available two-piece system termed ALT-R™ CRISPR/Cas9 Genome Editing System (IDT DNA, Coralville, IA). This system combines a custom CRISPR RNA (crRNA) for genomic specificity with an invariant trans-activating crRNA (tracrRNA) to produce gRNAs (Homanics, 2019). crRNAs were designed using the computational program CCTop/CRISPRator (Stemmer et al., 2015; Labuhn et al., 2018), which gauges candidate gRNAs for efficiency and specificity.

**FIGURE 2 |:**
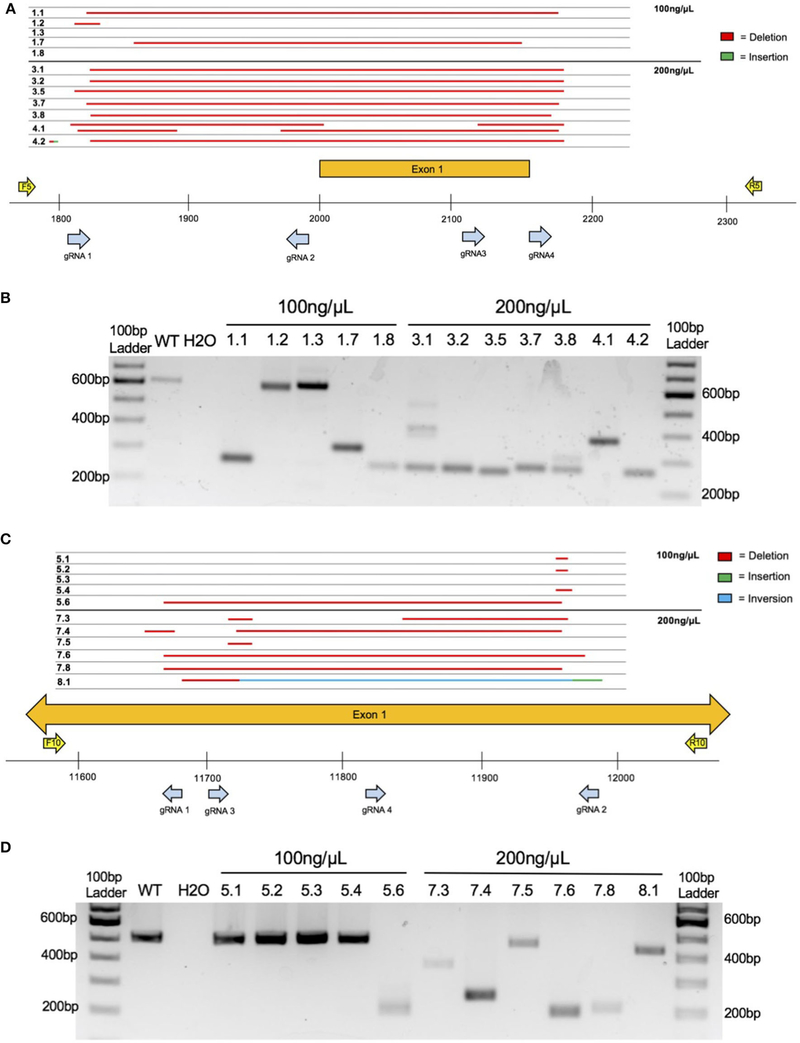
Embryo CRISPy TAKO genotypes for *4930425L21Rik* and *Gm41261*. **(A)** Sequence results for the major product(s) of TAKO embryos targeting gene *4930425L21Rik* electroporated with 100 and 200 ng/μL Cas9 protein. Deletions are shown in red. Sequence insertions are shown in green. Individual embryo numbers are presented on the left. The gRNAs and PCR primers used are shown as blue and yellow arrows, respectively. **(B)** Agarose gel electrophoresis of PCR amplicons for *4930425L21Rik* in embryos. Samples 1.1 through 1.8 were electroporated with 100 ng/μL Cas9 protein while samples 3.1 through 4.2 were electroporated with 200 ng/μL Cas9 protein. **(C)** Sequence results for the major product of TAKO embryos targeting gene *Gm41261* with 100 and 200 ng/μL Cas9 protein. Deletions are shown in red. Sequence insertions are shown in green. Sequence inversions are shown in blue. Individual embryo numbers are presented on the left. **(D)** Agarose gel electrophoresis of PCR amplicons for *Gm41261* in embryos. Samples 5.1 through 5.6 were embryos electroporated with 100 ng/μL Cas9 protein while samples 7.3 through 8.1 were electroporated with 200 ng/μL Cas9 protein.

**FIGURE 3 |:**
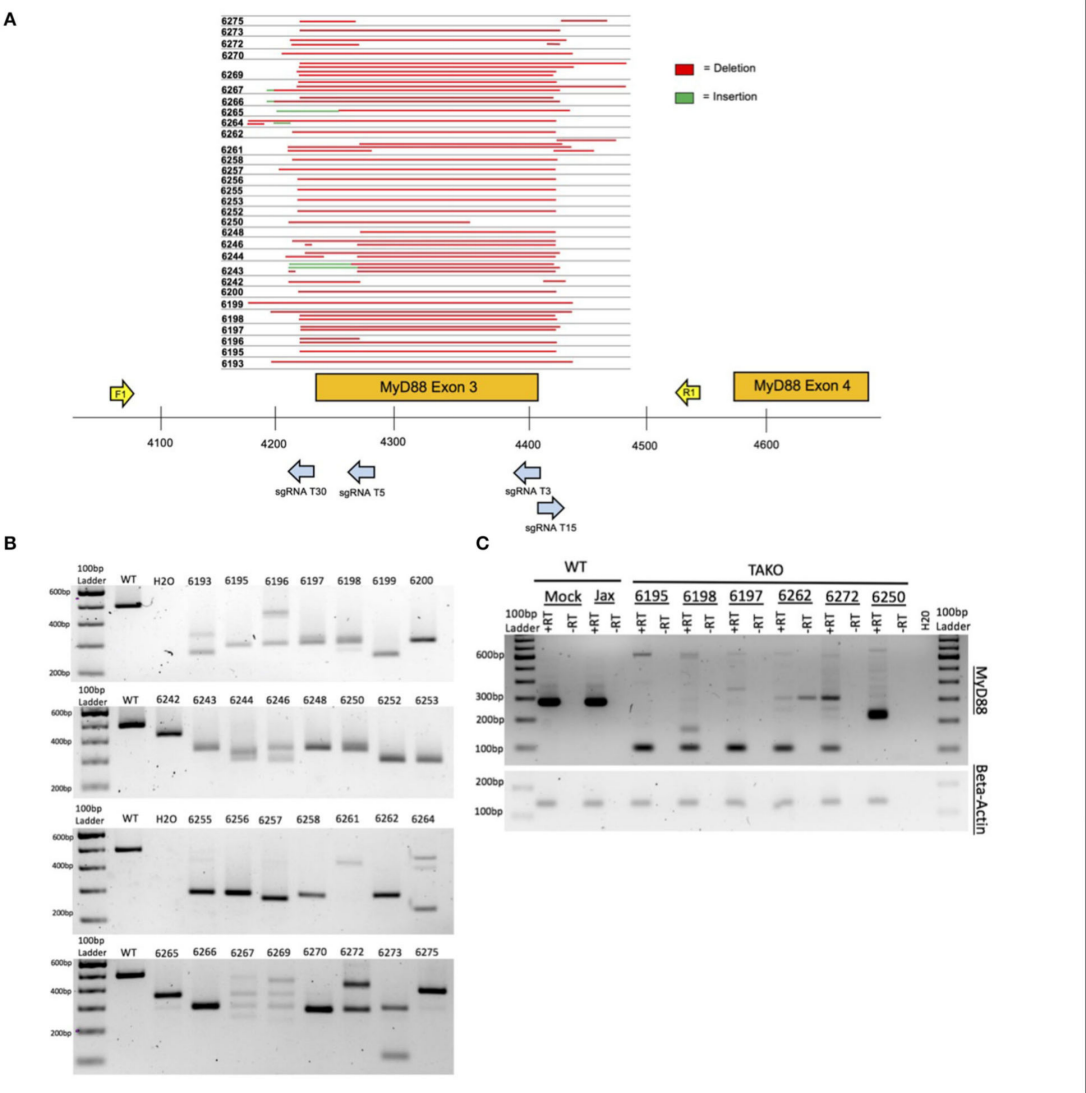
*In vivo* MyD88 CRISPy TAKO genotypes. **(A)** Sequencing results from mice selected for behavioral experimentation. CRISPR/Cas9 mutagenesis of MyD88 Exon 3 was performed and animals used for experimentation were sequenced to confirm successful deletion. Deletions are shown in red. Sequence insertions are shown in green. Individual animal tag numbers are presented on the left. The gRNAs and PCR primers used are shown as blue and yellow arrows, respectively. **(B)** PCR results from DNA of MyD88 CRISPy TAKO mice used for experimentation. Jax mouse used as WT control. **(C)** Random sample subset RT-PCR results from cerebellar brain tissue showing abnormal *MyD88* RNA transcripts in TAKOs. RT-PCR using Beta-Actin was used as an internal control.

Four crRNAs were used to target the ethanol-responsive lncRNA gene *4930425L21Rik* (see Supplementary Table 1 for gRNA target sequences). These four crRNAs bind within a 366 bp region that includes the putative promoter and first Exon (see Figure 2A). Similarly, four crRNAs were used to target the lncRNA gene *Gm41261* (see Supplementary Table 1 for gRNA target sequences). These four crRNAs bind within a 316 bp region of the first Exon (see Figure 2C). Four crRNAs were also selected for *MyD88* (see Supplementary Table 1 for gRNA target sequences) that bind within a 209 bp region that includes *MyD88* Exon 3 and flanking DNA (see Figure 3A). For each project, the four crRNAs were annealed separately with tracrRNA in a 1:2 molar ratio then combined into a single solution.

### CRISPR/Cas9 Mutagenesis

Female C57BL/6J mice were superovulated with 0.1 mL of CARD HyperOva (CosmoBio, #KYD-010) between 10 and 11 a.m., followed by 100 IU of human chorionic gonadotropin (Sigma, #CG10) 46–48 h later. Donor females were caged overnight with C57BL/6J males starting 4–6 h post-gonadotropin injection and allowed to mate. Embryos were harvested from oviducts between 9 and 10 a.m. the following morning, cumulus cells were removed using hyaluronidase, and embryos were cultured under 5% CO_2_ in KSOM medium (Cytospring, #K0101) for 1–2 h. Embryos were electroporated in 5 μL total volume of Opti-MEM medium (ThermoFisher, #31985088) containing 100 ng/μL of each sgRNA and 100 or 200 ng/μL Alt-R® S.p. HiFi Cas9 Nuclease V3 protein (IDT, #1081060) with a Bio-Rad Gene-Pulser Xcell in a 1 mm-gap slide electrode (Protech International, #501P1–10) using square-wave pulses (five repeats of 3 msec 25V pulses with 100 msec interpulse intervals). Two different concentrations of Cas9 protein were used to assess which produced greater mutagenesis in embryos targeting genes *4930425L21Rik* and *Gm41261*; only the 200 ng/μL concentration was used for MyD88 embryos. Electroporated embryos were placed back into culture under 5% CO_2_ in KSOM. For *4930425L21Rik* and *Gm41261*, embryos were cultured for three days until the morulea/blastocyst stage and subsequently analyzed for mutations. For MyD88, oneor two-cell embryos were implanted into the oviducts of plug-positive CD-1 recipients (20–40 embryos per recipient) that had been mated to a vasectomized male the previous night. Mock-treated controls were manipulated in parallel as described above, except that the electroporation mix lacked the *MyD88*-specific crRNAs (i.e., only tracrRNA and Cas9 protein were used).

### Genotyping

For *4930425L21Rik* and *Gm41261*, DNA was amplified from individual embryos using a Qiagen Repli-G kit (Qiagen, #150025) and subject to PCR genotyping under the following settings: 95°C for 5 min (1×); 95°C for 30 s, 60°C for 30 s, 72°C for 1 min (40×); 72°C for 10 min (1×). Primers for PCR amplification of *4930425L21Rik* and *Gm41261* are listed in Supplementary Table 1. PCR amplicons (WT = 613 and 506 bp, respectively) were analyzed by agarose gel electrophoresis and Sanger sequencing.

For *MyD88*, DNA was isolated from tail snips of MyD88 CRISPy TAKO and Mock-treated control offspring using Quick Extract (Lucigen, #QE09050). Primers for *MyD88* genotyping are listed in Supplementary Table 1. PCR amplicons (WT = 494 bp) were analyzed by agarose gel electrophoresis and Sanger sequencing.

### Subcloning

Samples that did not produce clear chromatograms were subcloned to identify allelic variants. The TOPO™ TA cloning kit (ThermoFisher Scientific, #K457501) was used according to manufacturers instructions, with slight modifications. Briefly, sample PCR product was incubated at room temperature for 15 min with TOPO reagents, then the TOPO vector mixture was incubated with chemically competent DH5α (ThermoFisher Scientific, #18265017) cells on ice for 30 min. Cells were then heat-shocked for 45 s in a 42°C water bath then immediately placed back on ice for 2 min. S.O.C. medium (ThermoFisher Scientific, #15544034) was added and cells were incubated in a bacterial shaker at 37°C for 90 min at 225 rpm. Cells were plated on kanamycin-resistent LB plates and incubated at 37°C for 16–18 h. Single colonies (*n* = 10 per sample) were collected and their DNA was used for PCR. Colonies that produced a single PCR band were then Sanger sequenced.

### RNA Preparation

Brain cerebellar tissue from one Mock treatment control (*n* = 1 male), one Jax control (*n* = 1 male), and 6 MyD88 mutants (*n* = 3 male, *n* = 3 female) were used for RT-PCR analysis. All mice were 11–12 weeks of age at time of sacrifice. Total RNA was isolated using TRIzol (Invitrogen, #15596018) according to the manufacturer’s protocol, and purified with a TURBO DNA-*free*™ Kit (Invitrogen, #AM1907). Total RNA was analyzed for purity and concentration using a Nanodrop Spectrophotometer (Thermo Scientific, Waltham, MA). One microgram of purified RNA was converted into cDNA using Superscript™ III First-Strand Synthesis System (Invitrogen, #18080051) with random hexamer primers. PCR primers were used that span from Exon 2 to Exon 4 (see Supplementary Table 1) of *MyD88*. A reaction that lacked reverse transcriptase was used as a negative control for each sample tested. RT-PCR amplicon size is 280 bp for WT, and 99 bp when Exon 3 is lacking.

### Behavioral Testing

All mice were moved into a reverse light-cycle housing/testing room (lights off at 10 a.m.) at 5 weeks of age and allowed to acclimate for 2–3 weeks before the start of experiments. Experiments were performed in the housing room (ethanol drinking) or an adjoining room (loss of the righting response (LORR), rotarod). Mice were group-housed 4 to 5 per cage based on genotype and sex. The same mice were sequentially tested on the rotarod, LORR, and drinking assays, with 4–7 days between assays.

### Drugs

Injectable ethanol solutions were prepared fresh daily in 0.9% saline (20%, v/v). Ethanol (Decon Laboratories, Inc.) was injected intraperitoneally (i.p.) at 0.02 mL/g of body weight.

### Rotarod

In order to assess ethanol-induced motor ataxia, mice were trained on a fixed speed rotarod (Ugo Basile, Gemonio, Province of Varese, Italy) at 11 rpm. Training was considered complete when mice were able to remain on the rotarod for 60 s. Following training, mice were injected with ethanol (2 g/kg, i.p.) and every 15 min mice were placed back on the rotarod and latency to fall was measured until mice were able to remain on the rotarod for 60 s.

### Loss of the Righting Response (LORR)

Sensitivity to the sedative/hypnotic effects of ethanol was determined using the LORR assay. Mice were injected with ethanol (3.5 g/kg, i.p.) and when mice became ataxic, they were placed in the supine position in V-shaped plastic troughs until they were able to right themselves three times within 30 s. LORR was defined as the time from being placed in the supine position until they regained their righting reflex. Body temperatures were maintained using a heat lamp throughout the assay.

### Two-Bottle Choice Every-Other-Day (2BC-EOD) Drinking

Mice were given access to ethanol (15%, v/v) and water for 24 h sessions every other day for 12 days starting at 12 p.m. Water alone was offered on off days. Purchased drinking bottles were 15 mL with 3.5-inch sipper tubes (Amuza, San Diego). The side placement of the ethanol bottles was switched with each drinking session to avoid side preference. Ethanol solutions were prepared fresh daily. Bottles were weighed before placement and after removal from the experimental cages. Empty cages with sipper bottles were used to control for leakage, and leakage amount was subtracted from amount consumed by the mice. The quantity of ethanol consumed, and total fluid intake, was calculated as g/kg body weight per 24 h. Preference was calculated as amount ethanol consumed divided by total fluid consumed per 24 h.

### Statistical Analysis

Statistical analysis was performed with GraphPad Prism (GraphPad Software, Inc., La Jolla, CA) for two-tailed Mann–Whitney test and two-way ANOVA (with mixed-effects analysis (i.e., when technical failures were present), multiple comparisons, and repeated measures when appropriate). Significant main effects were subsequently analyzed with Benjamini, Krieger, and Yekutieli two-stage linear step up procedure *post-hoc* analysis (Benjamini et al., 2006). Technical failures were approprietely removed from analysis.

The two control groups were first compared to one another; if no difference was found between control groups, these groups were pooled and tested against the MyD88 KO group. Graphs show control groups plotted separately even though they were analyzed together, unless noted otherwise. Because of well-known sex differences on the behaviors of interest, and because male and female mice were tested on separate days, each sex was analyzed separately. Statistical significance was defined as *p* < 0.05 and *q* < 0.05. All data are presented as mean ± S.E.M.

## RESULTS

### CRISPR/Cas9-Mediated Mutagenesis

Preliminary testing of the CRISPy TAKO method occurred *in vitro* using embryos electroporated at the one-cell stage, cultured until the blastocyst stage, then genotyped (Figure 2). To enhance CRISPR mutagenesis frequency, each gene was targeted simultaneously with four gRNAs that were tiled across a small section of the gene. In addition, we tested two concentrations of Cas9 protein (100 and 200 ng/μL) that were higher than the minimum amount we typically use (i.e., 50 ng/μL).

The first gene targeted was an unannotated ethanol-responsive gene, *4930425L21Rik*, using four gRNAs that span 366 bp of the putative promoter and first Exon (Figure 2A). Agarose gel electrophoresis of PCR amplicons that span the targeted locus indicated that three of five embryos tested at 100 ng/μL Cas9 had obvious indels whereas two embryos (#’s 1.2 and 1.3; Figures 2A,B) had amplicons that were grossly indistinguishable from the 613 bp WT control amplicon (Figure 2B). Sanger sequencing revealed #1.2 as heterozygous for WT and a 21 bp deletion (Figure 2A). At 200 ng/μL Cas9 protein, all seven embryos assessed were found to harbor deletions of varying sizes (Figures 2A,B). Thus, none of the embryos electroporated with 200 ng/μL Cas9 harbored WT amplicons that were visible on the agarose gel or detectable by amplicon bulk sequencing.

The second gene targeted was another unannotated ethanol-responsive gene, *Gm41261*. Four gRNAs spanning 316 bp within the putative first Exon were used (Figure 2C). Agarose gel electrophoresis of PCR amplicons that span the targeted locus indicated that one of five embryos tested at 100 ng/μL Cas9 had an obvious indel (#5.6; Figures 2C,D), whereas the other four of five embryos had amplicons that were indistinguishable from the 506 bp WT control amplicon (Figure 2D). Sanger sequencing revealed only one embryo (#5.3; Figures 2C,D) was homozygous WT, whereas the other four embryos harbored various small deletions (Figure 2C). At 200 ng/μL Cas9, all six embryos assessed were found to harbor deletions of varying sizes (Figures 2C,D). Although one embryo (#7.5) had a PCR product approximately the size of the WT amplicon (506 bp), Sanger sequencing revealed a 14 bp deletion. Sanger sequencing also revealed a sequence inversion in #8.1, along with a 16 bp insertion directly following the inverted sequence (Figure 2C). Thus, five of six embryos electroporated at 200 ng/μL Cas9 protein did not harbor detectable WT amplicons by agarose gel or amplicon bulk sequencing. Because the higher 200 ng/μL Cas9 concentration showed greater mutagenic activity in both *4930425L21Rik* and *Gm41261*, this concentration was utilized in targeting *MyD88*.

As proof-of-concept and to validate our method *in vivo*, we created MyD88 CRISPy TAKO mice. The four gRNAs were tiled across a 209 bp region of *MyD88* that included Exon 3 (Figure 3A). Exon 3 was targeted because prior traditional global MyD88 KO studies demonstrated that deletion of this Exon inactivates MyD88 (Hou et al., 2008) and imparts an ethanol behavioral phenotype (Wu et al., 2012; Blednov et al., 2017a,b).

Implantation of embryos electroporated on six different days with *MyD88* gRNAs yielded 54 offspring (*n* = 26 females, *n* = 28 males). Thirty-one offspring (*n* = 16 females, *n* = 15 males) were derived from electroporation of Mock-treated control embryos that were handled identically except that the crRNAs were omitted from the electroporation solution. All mice born from electroporated embryos were genotyped for gross indels at *MyD88* Exon 3 using endpoint PCR (data not shown). The 494 bp WT PCR amplicon was invariant and readily detectable in Jax and Mock-treated control samples as expected (Figure 3B; unpublished results). In stark contrast, 52 of 54 MyD88 KO mice displayed gross indels encompassing *MyD88* Exon 3 that were readily apparent following gel electrophoresis of PCR products (unpublished results). Out of the 54 MyD88 mutant mice created, a subset of mice were selected for behavioral phenotyping based on indel size (*n* = 30), and PCR results are shown in Figure 3B. The indels varied from animal to animal and most appeared to be deletions, as evidenced by the PCR products being ~50–300 bp smaller than the 494 bp WT amplicon. To more accurately characterize the mutations present, we sequenced the PCR products of the mutated mice selected for behavior (Figure 3A). As illustrated in Figures 3A,B, the mice used for phenotyping presented variable deletions mainly ranging from 200 to 300 bp. All deletions broadly encompassed Exon 3, therefore all mice were expected to manifest the same behavioral phenotypes. It is important to note that 100% of all crRNA-electroporated pups contained a *MyD88* indel. Based on the genotyping results, a subset of MyD88 CRISPy TAKOs (*n* = 15/sex) containing MyD88 inactivating mutations were selected for ethanol-related behavioral interrogation.

Cerebellar tissue from a random subset of MyD88 CRISPy TAKOs and controls were used for RT-PCR analysis using PCR primers that bind to Exons 2 and 4 to examine MyD88 mRNA. This analysis revealed the expected 280 bp fragment in both WT control samples (Figure 3C). In contrast, none of the six MyD88 CRISPy TAKO mice examined produced a fragment of this size. Five of the six samples produced a predominant band of ~99 bp as would be expected for *MyD88* mRNA that lacked Exon 3. One sample produced a major band of ~210 bp and may represent a splicing defect. Thus, MyD88 CRISPy TAKO mice are likely to be functional KOs.

### Ethanol-Induced Loss of Righting Response (LORR)

**FIGURE 4 |:**
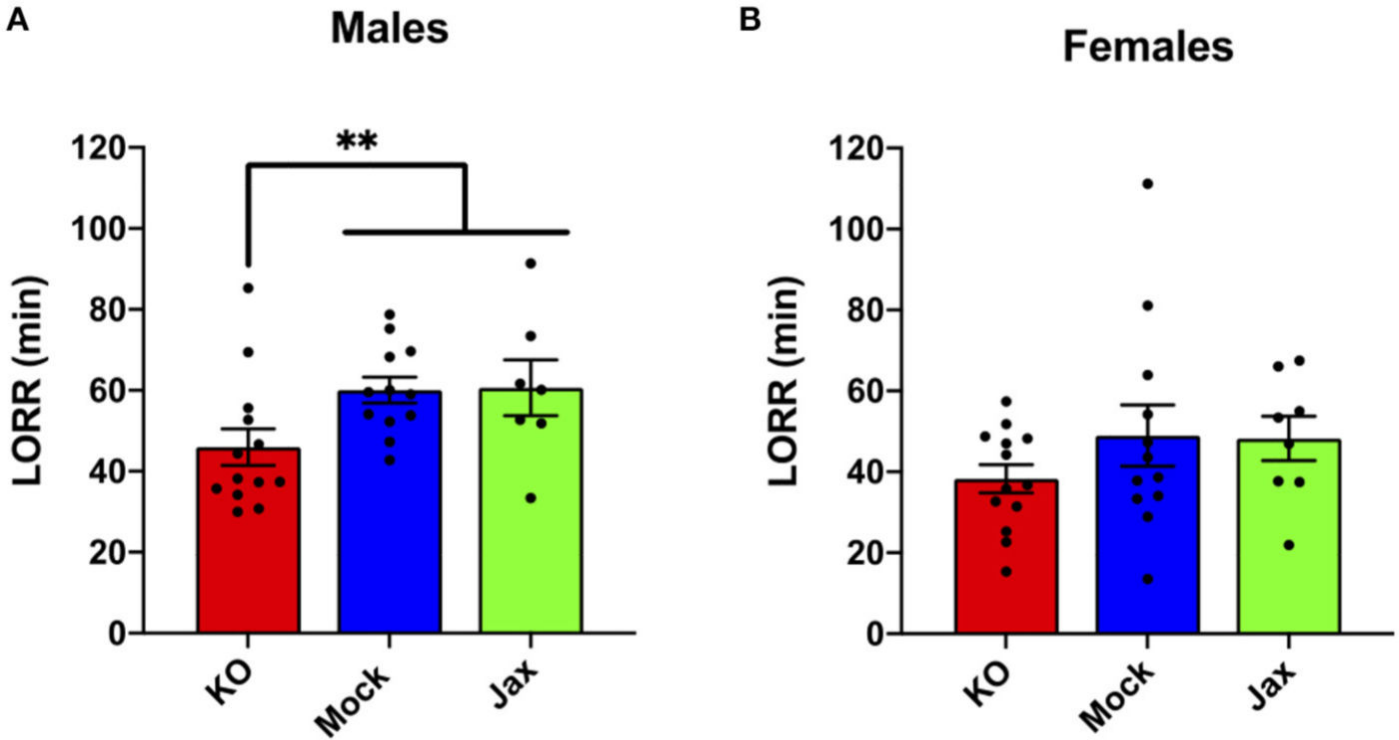
Duration of LORR induced by ethanol (3.5 g/kg, i.p.) injection in male **(A)** and female **(B)** MyD88 KOs, Mock controls and Jax controls. For both males and females, Mock controls and Jax controls did not differ and therefore were pooled and compared to same sex MyD88 KOs (data are plotted separately for completeness). KO males had reduced duration of LORR compared to the combined control group (**p < 0.01). For females, no difference was observed between MyD88 KOs and the combined control group. Values represent mean ± SEM. Data were analyzed using a two-tailed Mann–Whitney test.

No difference in ethanol LORR (3.5 g/kg, i.p.) was found between the Mock and Jax control groups for males or females (*p* = 0.9671 and *p* = 0.7345, respectively; Figure 4). Therefore, Mock controls and Jax controls were combined and compared to MyD88 KOs (for completeness, control results are plotted separately). Male MyD88 KOs exhibited a significant reduction in ethanol-induced LORR duration when compared to controls (*p* = 0.0068; Figure 4A). No difference was observed in females for ethanol-induced LORR (Figure 4B).

### Ethanol-Induced Motor Incoordination

**FIGURE 5 |:**
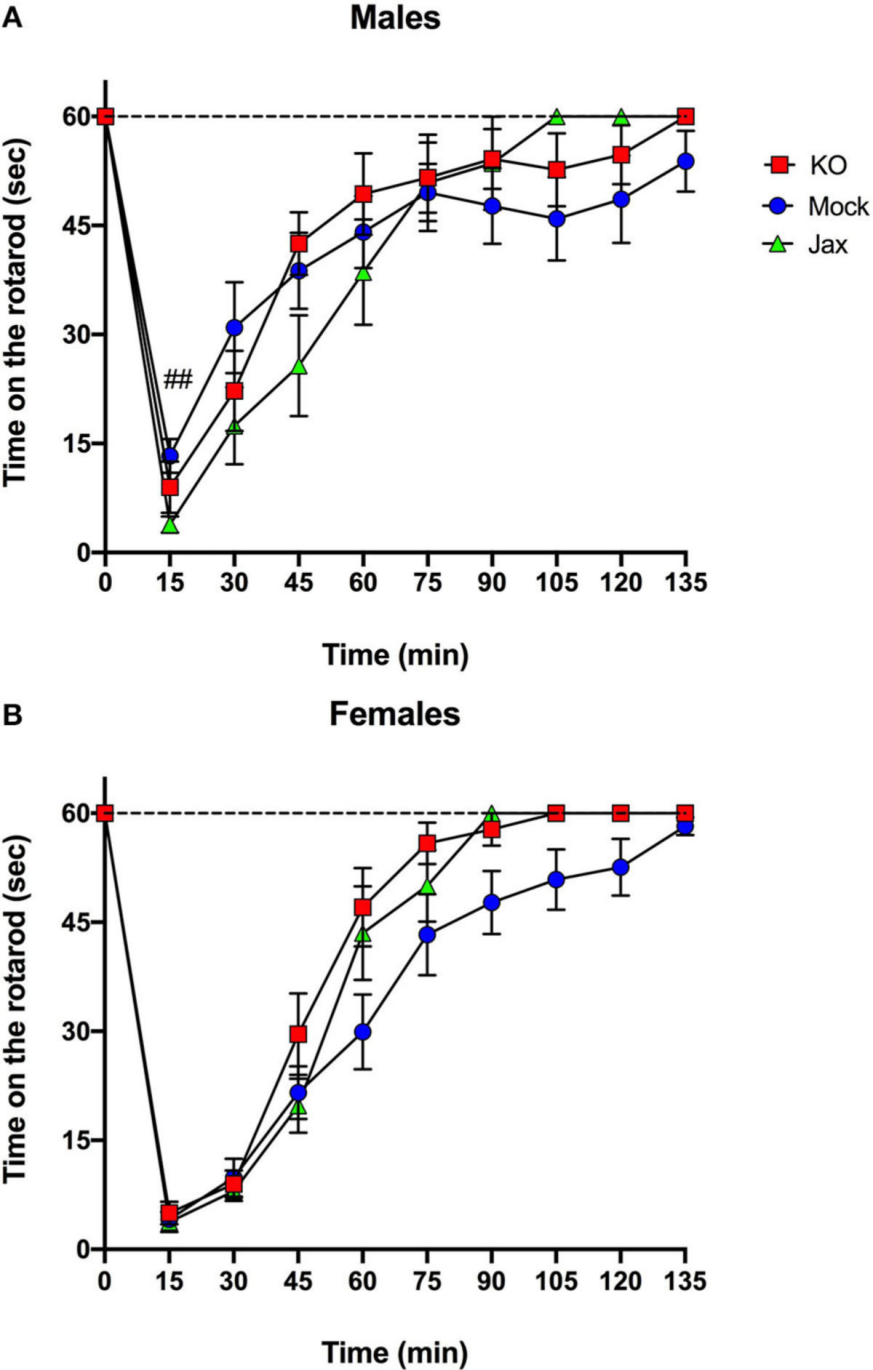
Recovery from ethanol-induced motor incoordination in MyD88 KO and control mice. **(A)** A significant time x genotype interaction was observed for male Mock controls and Jax controls. Therefore, control groups were not combined. Jax controls recovered more slowly compared to Mock controls at the 15min timepoint (##*q* < 0.01). **(B)** Female Mock controls and Jax controls did not differ and were combined for comparison to MyD88 KOs (data are plotted separately for completeness). *Post-hoc* analysis did not reveal any significant differences between groups at any time points. Data represent time in seconds on the rotarod after injection of ethanol (2 g/kg, i.p.). Values represent mean ± SEM. Data were analyzed by repeated-measures two-way ANOVA with multiple comparisons followed by Benjamini, Krieger, and Yekutieli *post-hoc* test.

The ataxic effects of an acute ethanol injection (2 g/kg, i.p.) were measured using a constant speed (11 rpm) rotarod test. For male mice, comparison of Mock and Jax controls showed a significant effect of time [*F*_(2.281, 41.05)_ = 36.41, *p* < 0.0001], and time x genotype [*F*_(9, 162)_ = 3.209, *p* = 0.0013], but no effect of genotype (Figure 5A). Because of the time x genotype interaction, control groups were not combined for this analysis. Repeated measures two-way ANOVA of all three groups revealed a significant effect of time [*F*_(2.474, 71.73)_ = 59.01, *p* < 0.0001], and an effect of time x genotype [*F*_(18, 261)_ = 1.964, *p* = 0.0120] but no effect of genotype (Figure 5A). *Post-hoc* comparisons revealed that male Mock control mice recovered more quickly than Jax controls at the 15 min timpoint (*q* = 0.0042).

For females, comparison of Mock and Jax controls revealed a significant effect of time [*F*_(2.775, 55.51)_ = 89.05, *p* < 0.0001], but no effect of genotype or time x genotype (Figure 5B). Therefore, the two control groups were combined (data plotted separately for completeness) and compared to MyD88 KOs. There was a significant effect of time [*F*_(2.664, 87.90)_ = 148.3, *p* < 0.0001], and genotype [*F*_(1, 33)_ = 4.721, *p* = 0.0371], but no effect of the time x genotype interaction (Figure 5B). *Post-hoc* analysis did not reveal any significant differences.

### Two-Bottle Choice Every-Other-Day (2BC-EOD) Drinking

**FIGURE 6 |:**
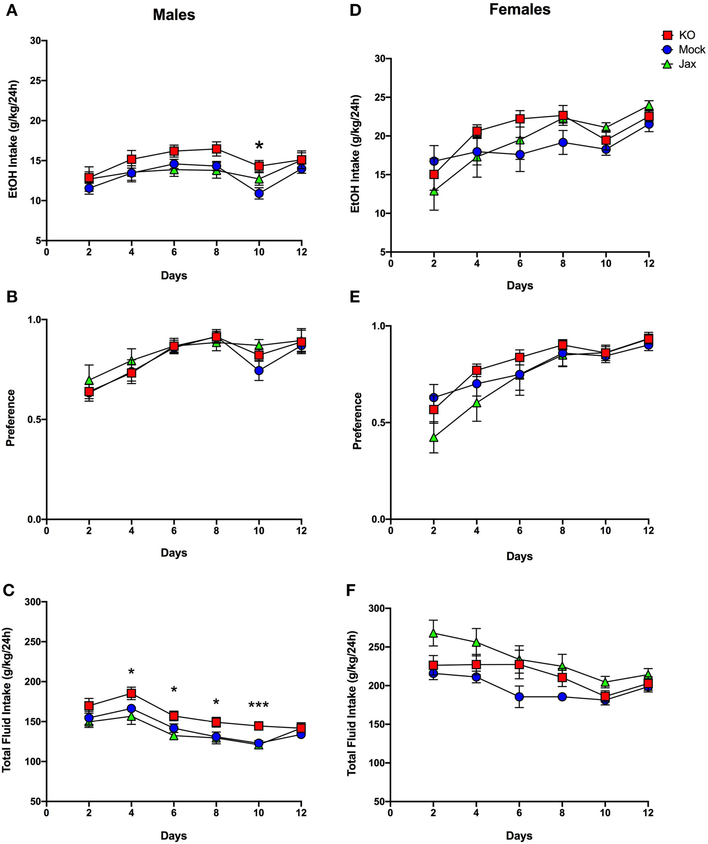
Two-bottle choice, every-other-day drinking in MyD88 KO, Mock control and Jax control mice. Left, males; right, females. **(A,D)** Ethanol (EtOH) intake (g/kg/24 h), **(B,E)** EtOH preference. **(C,F)** Total fluid intake (g/kg/24 h) in control (Jax C57BL/6J, *n* = 8; Mock-treatment control, *n* = 13–14) vs. mutant mice (*n* = 13–14 MyD88 KO). Values represent mean ± SEM. Data were analyzed by repeated-measures two-way ANOVA mixed-effects analysis with multiple comparisons followed by Benjamini, Krieger, and Yekutieli *post-hoc* tests (*q < 0.05 and ***q < 0.001 between MyD88 KO and combined controls.

Mice were tested for ethanol drinking using an intermittent every-other-day, two bottle free choice consumption assay over a period of 12 days. Mock and Jax control groups were first compared against each other; two-way ANOVA mixed-effects analysis was used for all 2BC-EOD statistical analyses. For males, there was a significant effect of time for ethanol intake [*F*_(3.333, 62.00)_ = 5.740, *p* = 0.0011], ethanol preference [*F*_(2.702, 50.27)_ = 10.85, *p* < 0.0001], and total fluid intake [*F*_(3.392, 63.09)_ = 17.98, *p* < 0.0001], but there was no effect of genotype or time x genotype interaction for any of these parameters (Figures 6A–C). Therefore, both Mock and Jax control groups were combined (data plotted separately for completeness) and compared to MyD88 KOs. Analysis of ethanol intake in males between the combined control group and MyD88 KO revealed a main effect of time [*F*_(4.123, 129.5)_ = 10.67, *p* < 0.0001] and genotype [*F*_(1, 32)_ = 4.850; *p* = 0.0350], but no interaction between the two (Figure 6A). *Post-hoc* analysis revealed that MyD88 KO males had significantly greater intake on day 10 (*q* = 0.0285) compared to controls. For preference in males, there was an effect of time [*F*_(3.365, 105.7)_ = 24.02, *p* < 0.0001] but no effect of genotype or time x genotype (Figure 6B). For total fluid intake in males, there was a main effect of time [*F*_(3.915, 122.9)_ = 36.79, *p* < 0.0001] and genotype [*F*_(1, 32)_ = 8.897, *p* = 0.0054], but no time x genotype interaction between MyD88 KO and controls (Figure 6C). *Post-hoc* analysis revealed significantly increased total fluid consumption in MyD88 KOs versus controls on days 4, 6, 8 (*q* = 0.0118), and day 10 (*q* = 0.0002) (Figure 6C).

In females, Mock and Jax control groups were first compared. There was a significant effect of time on ethanol intake [*F*_(2.412, 47.27)_ = 8.979, *p* = 0.0002] and ethanol preference [*F*_(2.626, 51.47)_ = 22.58, *p* < 0.0001], but no effect of genotype or time x genotype interaction on either parameter (Figures 6D,E). Therefore, both control groups were combined (data plotted separately for completeness) and compared to MyD88 KOs. For ethanol intake, females showed a main effect of time [*F*_(2.632, 86.85)_ = 12.50, *p* < 0.0001] but not genotype or time x genotype interaction (Figure 6D). Similarly, for ethanol preference a significant effect of time [*F*_(3.317, 109.5)_ = 29.10, *p* <0.0001] but not genotype and time x genotype interaction was observed (Figure 6E). Thus, ethanol intake and preference did not differ between female MyD88 KOs and the combined control group.

Comparing total fluid intake in female Mock and Jax controls revealed significant main effects of time [*F*_(2.818, 55.22)_ = 9.800, *p* < 0.0001] and genotype [*F*_(1, 20)_ = 10.41, *p* = 0.0042], but no time x genotype interaction (Figure 6F). Therefore, for female total fluid intake, control groups were not combined and each genotype was considered separately. This analysis revealed a significant effect of time [*F*_(2.650, 84.79)_ = 11.20, *p* < 0.0001] and genotype [*F*_(2, 33)_ = 4.221, *p* = 0.0223] (Figure 6F), but no time x genotype interaction. *Post-hoc* analysis did not reveal any significant differences.

## DISCUSSION

The current study reports on a CRISPR/Cas9-mediated mutagenesis protocol that is suitable for rapid screening for the phenotypic effects of gene KO *in vivo*. Traditional CRISPR mouse KO procedures require three generations of animal breeding and genotyping, which is time consuming and expensive. In contrast, with the CRISPy TAKO protocol described here, first generation gene-targeted F0 mice can be rapidly produced and screened for phenotypic effects. Although individual F0 mice harbor a variety of mutant alleles for the gene of interest, careful project design ensures that each F0 animal is functionally equivalent, i.e., a gene KO. F0 animals can be directly screened for phenotypes of interest. If no phenotype is detected, the gene is rapidly eliminated from further consideration. If an interesting phenotype is observed, a F0 animal can be bred as in traditional approaches to establish a true breeding line that can first be tested to confirm the phenotype. This will ensure rigor and reproducibility in the experimental pipeline. Subsequently, the line can be maintained long-term and more detailed, rigorous mechanistic studies can be conducted. The CRISPy TAKO approach can save valuable time and minimize animal numbers and financial resources.

There are several keys to the success of this approach. First, we use embryo electroporation to facilitate genetic modification of a large number of animals with minimal effort. Large numbers of embryos (*n* = 30–50) can be simultaneously transfected with CRISPR reagents at a very high efficiency (Chen et al., 2016; Hashimoto et al., 2016; Wang et al., 2016; Wefers et al., 2017; Modzelewski et al., 2018). This avoids the limiting bottleneck of directly injecting each individual embryo. Second, achieving a very high level of indel formation that ablates function of the gene of interest in each animal is critical. We observed that 52 of 54 of animals harbored inactivating indels, while the other two harbored smaller mutations that may or may not have been inactivating (mice were deemed insuitable for experimentation and therefore the mutations were not sequenced). To achieve this high KO efficiency, we simultaneously utilized four gRNAs that targeted a small, functionally important portion of the gene of interest. In other experiments, we have observed that the mutagenesis efficiency of a single gRNA is highly variable. Simultaneous use of two gRNAs tends to increase mutagenesis efficiency. We reasoned that an even higher number of gRNAs tiled across a small but functionally important part of a gene would result in even higher efficiency. We are unsure if four is the optimal number of gRNAs, but this should be rigorously explored in future studies. For *in vivo* proof-of-concept, we focused on a small, single Exon of the *MyD88* gene that results in a null allele when disrupted (Hou et al., 2008; Wu et al., 2012; Blednov et al., 2017a,b). This approach should also work by targeting the promoter or any region that is critical for function of the gene and/or gene product. It should also be noted that we observed that 200 ng/μL Cas9 protein in the electroporation mix produced a much higher rate of indel formation compared to 100 ng/μL. While 200 ng/μL is 4× the minimum amount we typically use in our lab for most CRISPR embryo electroporation experiments, this amount is less than that reported in the literature (Wang et al., 2016; Wefers et al., 2017).

Unbeknownst to us at the time we conducted these experiments, a similar multi-gRNA CRISPR strategy termed C-CRISPR had been previously reported (Zuo et al., 2017). Although the primary application of the C-CRISPR method was to create KO mice and non-human primates with reduced levels of mosaicism, the authors also recognized the usefulness of such an approach for phenotypic screens in F0 animals. Together, the current study and that of Zuo et al. (2017) demonstrate that multi-gRNA approaches are remarkably robust across laboratories and target loci. Despite the similarities, CRISPy TAKO and C-CRISPR differ in a few subtle, but possibly important ways. To introduce CRISPR reagents into embryos, we used electroporation whereas C-CRISPR used microinjection of individual embryos. With electroporation, large numbers of embryos can be transfected with minimal time and effort, and electroporation does not require expensive embryo microinjection equipment and embryo micromanipulation skills. Secondly, we used high fidelity Cas9 protein whereas C-CRISPR used Cas9 mRNA. Lastly, as described below, we also tested and validated the use of animals procured directly from a vendor as a control group for comparison to F0 KO animals in phenotypic screens. These subtle differences provide the CRISPy TAKO approach with significant savings of time and financial resources.

The CRISPy TAKO approach could be further streamlined and throughput increased if control animals for comparison could be procured directly from a vendor. However, it is conceivable that the *in vitro* embryo manipulation / CRISPR electroporation procedure could introduce some unknown variable that could impact the phenotype of interest regardless of the gene targeted for modification. Therefore, we compared phenotypes of control animals procured directly from JAX with isogenic controls that were produced in-house, in parallel to the MyD88 TAKOs. This in-house control group was created using procedures that were identical to those used to create MyD88 TAKOs except that crRNAs were omitted from the electroporation reactions. We observed near complete concordance between these Mock controls and Jax control animals for the behavioral phenotypes of interest. We only observed a subtle female-specific difference in total fluid consumption in the 2BC-EOD assay (Figure 6F) and a male-specific genotype x time interaction on the rotarod (Figure 5A). We conclude that mice purchased directly from a vendor can be used as a control group for screening CRISPy TAKO mice for behavioral alterations provided the controls are the same genetic background as those animals that served as embryo donors. Using a single vendor-derived control group will substantially increase throughput and reduce expenses.

As proof-of-concept of the CRISPy TAKO approach, we focused on *MyD88* as a candidate gene. We sought to functionally validate our approach by comparing behavioral phenotypes observed with those previously reported for global MyD88 KO mice produced using traditional gene-targeting technology, which displayed robust alterations in ethanol-induced behavioral responses and ethanol drinking behavior (Wu et al., 2012; Blednov et al., 2017a,b). Overall, similar behavioral results were observed between traditional MyD88 KOs and MyD88 CRISPy TAKOs (Table 1). Consistent with previous findings, the MyD88 KO females show faster recovery time from ethanol’s incoordination effects (Figure 5B), but contrary to those studies, no difference between male MyD88 KOs and controls is reported here (Figure 5A). Also consistent with previous reports, albeit with a milder effect size (Blednov et al., 2017b), MyD88 KO males had greater consumption of ethanol than controls (Figure 6A). However, KO males in the present study did not have a difference in preference when compared to controls (Figure 6B), but had significantly increased total fluid intake compared to controls (Figure 6C), suggesting these male mice drink more fluid in general, and it is not specific to ethanol. Altered total fluid intake in MyD88 KO females compared to controls (Figure 6F) is consistent with the published literature (Blednov et al., 2017b).

It is unclear if the results presented here show a milder phenotype than those previously reported (Wu et al., 2012; Blednov et al., 2017a), or if these differences are simply due to experimental variation that is common in behavioral studies between labs, facilities, and universities (Crabbe et al., 1999; Wahlsten et al., 2006). Although all studies were conducted using C57BL/6J mice, the current study utilized mice sourced directly from JAX, whereas Blednov et al. (2017a,b) used mice sourced from JAX that were bred in-house for an unspecified number of generations. It is also possible that the CRISPy TAKO approach is slightly less sensitive than the traditional KO approach for detecting phenotypic changes. The most likely explanation for such a possibility is that F0 CRISPR mice are often mosaic and it is conceivable that tail DNA genotyping is not reflective of the genetic changes that occur in the brain of the mutant animals. It is possible that some WT MyD88 is expressed in the brain of some F0 animals, however this is unlikely because RT-PCR analysis of a subset of MyD88 TAKO mice did not have WT bands (Figure 3C). The approach described here should be very useful for a first pass screening method to identify genes with a large effect on a phenotype of interest. The usefulness of this approach for detecting subtle genotypic differences requires further evaluation.

One limitation of the approach as outlined is the potential for off-target effects of CRISPR mutagenesis. This approach uses multiple gRNAs simultaneously along with a relatively high concentration of Cas9 protein, both of which could lead to off-target effects. Although off-target effects were not examined in this study, they are unlikely to explain the phenotypic changes we observed. The main behavioral phenotypes observed in the MyD88 CRISPy TAKO mice are the same as those observed in MyD88 global KOs that were produced using traditional, non-CRISPR gene targeting techniques. Furthermore, several studies have reported that off-target effects in CRISPR/Cas9 animals is minimal with careful selection of gRNAs as done in the present study (Iyer et al., 2018; Willi et al., 2018; Dong et al., 2019). We also used a high fidelity Cas9 protein variant to minimize the potential for off-target effects. Lastly, an extensive analysis of gene KO mice and non-human primates that were produced by CRISPR mutagenesis with multiple gRNAs found no significant off-target effects (Zuo et al., 2017).

In summary, we propose using the CRISPy TAKO approach for rapidly screening large numbers of genes *in vivo* to identify those that have large effects on a phenotype of interest. Once such a gene is identified, an individual animal that harbors a confirmed KO allele should be mated to establish a true breeding mutant KO line. A true breeding line will be useful for future studies to (a) confirm the phenotype of interest, (b) to test for and rule out the potential impact of off-target mutations, (c) to enable the rigorous testing of control and KO littermates derived from heterozygous matings, and (d) to provide an unlimited source of uniform animals for further, in-depth analyses and long-term line maintenance.

We conclude that the CRISPy TAKO method can be used for efficient, moderate throughput, *in vivo* screens to identify genes that impact whole animal responses when ablated. This method avoids the extensive animal breeding, time, and resources required with traditional CRISPR animal KO approaches. This method should find widespread use in studies where moderate to large numbers of genes must be rapidly screened for effects that cannot be interrogated *in vitro*, such as whole animal behavioral responses.

## Supplementary Material

Suppl Table 1

## Figures and Tables

**TABLE 1 |:** Comparison of MyD88 cohort results with previous findings.

	CRISPy TAKO		Previous Reports	
	Males	Females	Males	Females
Rotarod	No change	Faster recovery	Faster recovery (Wu et al., 2012; Blednov et al., 2017a)	Faster recovery (Blednov et al., 2017a)
Ethanol Loss of Righting Response	Reduced duration	No change	Reduced duration (Wu et al., 2012; Blednov et al., 2017a)	Reduced duration (Blednov et al., 2017a)
2BC-E0D (15% v/v)	Increased ethanol drinking	Reduced total fluid intake	Increased ethanol drinking and preference. Reduced total fluid intake (Blednov et al., 2017b)	Reduced total fluid intake (Blednov et al., 2017b)

## Data Availability

The raw data supporting the conclusions of this article will be made available by the authors, without undue reservation.
