# Supplementary material for: CRISPR Turbo Accelerated KnockOut (CRISPy TAKO) for Rapid in vivo Screening of Gene Function
Source: Front Genome Ed. 2020 Oct 21;2:598522. doi: 10.3389/fgeed.2020.598522 (PMC7889042; doi:10.3389/fgeed.2020.598522)
Supplement: Supplementary file 1 [file Table_1.docx]

**Supplemental Table 1.** gRNA target sites and PCR primer sequences. All sequences are written in a 5’ to 3’ orientation. Note: the underlined sequence in each gRNA target site is the protospacer adjacent motif.

| **Name** | **Sequence** |
| --- | --- |
| *4930425L21Rik* #1 gRNA | ﻿AGACACTAATATTGCAGACG AGG |
| *4930425L21Rik* #2 gRNA | ﻿TTATTTTTCTGCAAGGGGTT GGG |
| *4930425L21Rik* #3 gRNA | ﻿ACACTATCGACCTAATAGCT AGG |
| *4930425L21Rik* #4 gRNA | ﻿ATTTTAAACCCTCTGTTACT TGG |
| *Gm41261* #1 gRNA | ﻿CAATTTGCAATTCTCTTCCA GGG |
| *Gm41261* #2 gRNA | ﻿AGAATAAACAGGTGTGACGG TGG |
| *Gm41261* #3 gRNA | ﻿CTTATCAGGTCTTTGATCAG AGG |
| *Gm41261* #4 gRNA | ﻿GGTCTTTTACTTTTCTCTTT AGG |
| MyD88 T3 gRNA | ﻿CCTTTTCTCAATTAGCTCGC TGG |
| MyD88 T5 gRNA | ﻿GCACAAACTCGATATCGTTG GGG |
| MyD88 T15 gRNA | ﻿AGGTTGGTTAAACATCTAAG AGG |
| MyD88 T30 gRNA | ﻿GGCGTTTGTCCTGAGGACAG GGG |
| *4930425L21Rik* F5 PCR primer | ﻿GTGTCCAGCATTGTGCCAAG |
| *4930425L21Rik* R5 PCR primer | ﻿TCTAAAAGGGGCCCTCCAGT |
| *Gm41261* F10 PCR primer | ﻿CTCACCAAAATTCAACCTGGAG |
| *Gm41261* R10 PCR primer | ﻿GCTTCAGAGCTCACTGGTGT |
| MyD88 F1 PCR primer | CCGGGATTTCATCTGGGAGG |
| MyD88 R1 PCR primer | ﻿ACTGCGGTGACTTCCTTCAG |
| MyD88 F2 PCR primer | GGTGGCCAGAGTGGAAAGCAGTGTCCC |
| MyD88 R2 PCR primer | GAAACAACCACCACCATGCGGCGACA |
